# Patient Follow-Up After Participating in a Beach-Based Skin Cancer Screening Program

**DOI:** 10.3390/ijerph9051836

**Published:** 2012-05-10

**Authors:** Mary L. Greaney, Elaine Puleo, Alan C. Geller, Stephanie W. Hu, Andrew E. Werchniak, Susan DeCristofaro, Karen M. Emmons

**Affiliations:** 1 Center for Community-Based Research, Dana-Farber Cancer Institute, Boston, MA 02215, USA; Email: Karen_M_Emmons@dfci.harvard.edu; 2 Department of Public Health, University of Massachusetts, Amherst, MA 01003, USA; Email: epuleo@schoolph.umass.edu; 3 Department of Society, Human Development & Health, Harvard School of Public Health, Boston, MA 02115, USA; Email: ageller@hsph.harvard.edu; 4 New York University Medical Center, New York, NY, 10016, USA; Email: stephwhu@gmail.com; 5 Department of Dermatology, Brigham and Women’s Hospital, Boston, MA 02115, USA; Email: awerchniak@partners.org; 6 Dana-Farber Cancer Institute, Boston, MA 02215, USA; Email: Susan_DeCristofaro@dfci.harvard.edu

**Keywords:** cancer screening, skin cancer prevention, skin examinations, sun protection

## Abstract

Many skin cancer screenings occur in non-traditional community settings, with the beach being an important setting due to beachgoers being at high risk for skin cancer. This study is a secondary analysis of data from a randomized trial of a skin cancer intervention in which participants (n = 312) had a full-body skin examination by a clinician and received a presumptive diagnosis (abnormal finding, no abnormal finding). Participants’ pursuit of follow-up was assessed post-intervention (n = 283). Analyses examined: (1) participant’s recall of screening results; and (2) whether cognitive and behavioral variables were associated with follow-up being as advised. Just 12% of participants (36/312) did not correctly recall the results of their skin examination. One-third (33%, 93/283) of participants’ follow-up was classified as being not as advised (recommend follow-up not pursued, unadvised follow-up pursued). Among participants whose follow-up was not as advised, 71% (66/93) did not seek recommended care. None of the measured behavioral and cognitive variables were significantly associated with recall of screening examination results or whether follow-up was as advised. Research is needed to determine what factors are associated with follow-up being as advised and to develop messages that increase receipt of advised follow-up care.

## 1. Introduction

Skin cancer is the most commonly diagnosed cancer in the United States, and unprotected exposure to the sun’s ultraviolet (UV) rays is a major contributing factor to the development of approximately two-thirds of all skin cancer cases [1]. Each year more than a 3.5 million new cases of non-melanoma skin cancers (basal cell carcinoma and squamous cell carcinoma) occur [2]. While seldom fatal, advanced non-melanoma skin cancers can cause severe disfigurement and morbidity. In addition, rates of melanoma, the most serious form of skin cancer, are increasing, particularly among young white women and older white men [3]. In 2011 an estimated 70,230 incident cases of melanoma were diagnosed in the United States [4]. Skin cancer screening has the potential to reduce death rates, but screening rates remain low [5,6].

Although the efficacy of screening for skin cancer has not been evaluated in a randomized clinical trial, the high curability of cancers like melanoma with early surgical excision and the low risk associated with the non-invasive screening procedure argue for the potential utility of screening [7]. There is, however, a lack of consistency with regard to skin cancer screening recommendations for the general population in the United States. For example, the U.S. Preventive Services Task Force states that there is insufficient evidence about the benefits of a whole-body skin examination conducted by primary care clinicians for early detection of cutaneous melanoma [8]. The American Cancer Society recommends that physicians periodically screen individuals 20 years of age or older for skin cancer and that the screening can be incorporated into general health examinations [9] while the American Academy of Dermatology (AAD) recommends yearly screening [10].

From its inception in 1985, more than two million free screenings have been conducted as part of the AAD national screening programs [11]. These free screening programs have resulted in a greater proportion of early-stage diagnosis compared with population-based results from the Surveillance, Epidemiology and End Results (SEER) database [11] and at least one-half of all screenees report that they would not have sought attention for suspicious lesions if it were not for the AAD programs [12]. Approximately one-third of the AAD’s screening programs occur in community-based settings rather than private office settings [13]. Skin cancer screenings are also commonplace in European nations and occur in multiple settings [14,15,16,17,18,19]. One important setting for skin cancer screening is the beach, as it can attract high risk individuals.

Screening programs are only effective when participants understand the examination results and seek prompt follow-up when warranted. This is of particular concern in the context of community-based screenings that count on participants to seek advised follow-up rather than relying on clinic staff to conduct follow-up telephone calls with participants who have a positive screening diagnosis in a clinical setting. Same day assistance with scheduling needed follow-up appointments may be offered to individuals attending community-based screenings who do not have a dermatologist, or insurance coverage. However, if participants do not accurately recall or comprehend their screening finding or do not take the results of the examination seriously, it is unlikely that needed follow-up action will be pursued. It is important to understand what behavioral and cognitive factors are associated with appropriate follow-up to community-based skin cancer screenings, as this understanding may provide important intervention targets that can used to design messages to promote pursuit of appropriate follow-up. Thus, in the beach-setting, we examined: (1) participant’s recall of screening results; and (2) if cognitive and behavioral variables predict whether follow-up was as advised.

## 2. Methods

Data are from a randomized controlled trial designed to promote the early detection and prevention of skin cancer that took place at public beaches in low-to-middle income communities [20]. The study was approved by the Institutional Review Board (IRB) at Harvard School of Public Health. This present study includes all participants recruited in 2007 from two study beaches where participants received a full-body skin examination. Study staff visited each beach on 3-4 designated days randomly assigned in advance with the intervention being offered on an equal number of weekends *vs*. weekdays. Recruitment took place in the beach parking lot and on the beach itself: study staff approached beachgoers and provided a brief description about the study, and invited them to join the study.

Eligibility requirements included being aged 18+; being able to speak and read English; and providing informed consent. Following consent, participants completed a survey (survey 1), took a sun safety quiz, and received a brief educational session that provided basic information about skin cancer knowledge, including signs and symptoms of common skin cancers, and sun protection information from study staff. A clinician (dermatologist, dermatology nurse practitioner) conducted a skin examination in a mobile health van and recorded findings. The clinician conducting the screening checked off the findings on a standard form, and participants received a presumptive diagnosis (no finding or abnormal finding) with a record of these results to discuss with their personal physician. Follow-up care was advised when warranted and its importance emphasized, and participants needing referrals were given the AAD’s toll-free number. Immediately afterwards, participants completed survey 2. The final survey (survey 3) was completed via mail at the end of summer. The baseline sample (n = 312) completed both surveys 1 and 2, and 90% of the baseline sample (n = 283) completed the final follow-up survey (survey 3). The loss to follow-up did not differ by exam results. Analyses were restricted to Caucasians due to their higher risks of skin cancer [21,22,23]. 

### 2.1. Measures

#### 2.1.1. Outcome Variables

*Screening diagnosis*. Findings from the skin examination were categorized as abnormal (actinic keratosis, basal cell cancer, squamous cell cancer, dysplastic nevus, melanoma) or not.

*Recall of presumptive diagnosis*. On survey 2, participants self-reported if the examination revealed suspicious moles, skin cancers, or pre-cancers, with a yes response to any of these conditions indicating that the participants believed that their exam revealed an abnormal finding. Responses were compared to the clinicians’ findings to determine concordance.

*Follow-up after skin examination*. On survey 3, participants reported if they had seen a physician for a subsequent skin examination and/or had any related dermatologic procedures. Follow-up was deemed as advised if: (1) participants sought recommended follow-up; or (2) those who were not advised to seek additional care reported no follow-up care. Follow-up was categorized as not as advised if: (1) participants with an abnormal finding did not seek follow-up; or (2) unnecessary follow-up care was pursued.

#### 2.1.2. Predictor Variables

Survey 1 assessed participants’ age, gender, race/ethnicity, and education, previous diagnosis of melanoma, history of atypical or unusual moles, and if their skin burned easily while in the sun.

#### 2.1.3. Behavioral and Cognitive Variables

*Sun protection behaviors*. Using measures widely used in the literature, participants reported frequency of wearing a hat, applying sunscreen (SPF15+), and limiting time in the sun between 10 AM and 4 PM during the past month (dichotomized: never, rarely, or sometimes *vs*. often, always).

*Skin screening behaviors*. Participants reported if they had conducted a careful skin self-examination, including examining their back during the past month prior to completion of the survey (yes, no) [12]. Additionally, participants reported if they had ever previously had a full skin examination conducted by a dermatologist (yes, no).

*Cognitive variables*. Participants rated their perceived risk of skin cancer (below average, average, above average), and assessed the sun damage to their skin (a small amount, a moderate amount, a lot).

### 2.2. Statistical Analysis

We used kappa statistics to determine if the clinician recorded outcome and patient recall of the results were concordant, and bivariate analyses to examine associations between the predictor variables and concordance of findings and participants’ recall of results. Similar analyses examined associations between the predictor variables, appropriateness of follow-up, and receipt of care by those advised to seek it. Analyses were conducted using SAS Version 9.3 [24].

## 3. Results

The majority of the sample (n = 312) was middle-aged, female, and a third reported less than a high school diploma. One-third had had a full-body dermatologic skin cancer examination prior to joining the study (see Table 1).

**Table 1:** Demographics, skin cancer risk factors and baseline behavioral and cognitive variables for individuals participating in a beach-based skin cancer screening in Massachusetts in 2007 (n = 312) and by whether follow-up was as advised (n = 283). ^a^

		Entire sample	Follow-up	
			As advised ^b^ (n = 190)	Not as advised ^c^ (n = 93)
		Mean (SD) or % (n)	Mean (SD) or % (n)	Mean (SD) or % (n)
**Demographic variables**				
Mean age (in years)		52.4 (SD = 15.6)	53.0 (SD = 15.3)	54.1 (SD = 15.3)
Sex (% female)		56 (173)	60.0 (114)	51.6 (48)
Education				
	High school diploma or less	36 (111)	35 (66)	39 (36)
	Some college	29 (91)	29 (53)	31 (29)
	College degree or more	35 (109)	37 (70)	30 (28)
**Skin cancer risk factors**				
History of melanoma (% yes)		4 (10)	6 (6)	4 (4)
Fair/very fair complexion (% yes)		59 (184)	58 (110)	66 (61)
Burn easily in the sun (% yes)		47 (146)	44 (86)	48 (45)
**Behavioral variables**				
**Sun protection behaviors** (% often/always)				
Wear hat		30 (95)	28 (54)	30 (28)
Limit time in sun		22 (68)	22 (42)	25 (23)
Use SPF 15 sunscreen		36 (112)	37 (71)	35 (33)
**Skin screening practices**				
Perform skin self examination past month (% yes)		31 (96)	28 (53)	33 (30)
Had full-body dermatologist skin examination (% yes)		32 (101)	28 (55)	38 (35)
**Cognitive variables**				
Perceived sun damage in lifetime (% none/small amount)		42 (131)	41 (80)	40 (37)
Perceived skin cancer risk				
	Below average risk	21 (65)	19 (37)	23 (21)
	Average risk	56 (175)	61 (115)	53 (49)
	Higher than average risk	23 (71)	19 (37)	25 (23)
Notes: ^a^ Due to rounding and/or missing data percent totals may not =100; ^b^ Follow-up was deemed appropriate if: (1) participants sought advised follow-up care; or (2) participants who were not advised to see additional care reported no follow-up care; ^c^ Follow-up was categorized as not as advised if: (1) participants with an abnormal finding did not seek follow-up; or (2) unnecessary follow-up care was pursued.				

Immediately after their skin-examination, most participants (88%, 276/312) accurately recalled their examination results, and there was no difference in recall of results based on presumptive diagnosis (abnormal finding, no abnormal finding). Sixteen participants reported an abnormal finding where none had been found and 20 reported no abnormal finding when, in fact, there had been one. The agreement between the recorded results and participant’s recall of the results was acceptable (kappa statistic = 0.64). None of the behavioral and cognitive variables were associated with accurate recall of the screening examination results in the bivariate models, and results did not change using continuous variables (data not shown).

Two-thirds (67%, 190/283) of all respondents’ follow-up to their skin examination was classified as advised and 33% (93/283) was classified as not as advised. Of those whose follow-up was not as advised, 29% (27/93) sought unnecessary care and 71% (66/93) did not seek advised follow-up care.

In total, 33% (104/312) of examinations revealed an abnormal finding. As seen in Table 2, pursuit of follow-up was lowest among those with screening findings of actinic keratoses (74% (26/35), did not seek follow-up care) and dysplastic nevus (73% (19/27) did not seek follow-up care). There were no significant associations between the behavioral (sun protection behaviors, skin examination behaviors) and cognitive variables (perceived risk of skin cancer, perceived sun damage to skin) and whether follow-up was as advised. Furthermore, there were no significant associations between the measured behavioral and cognitive variables and whether participants whose examination revealed an abnormal finding pursued recommended follow-up (data not shown).

**Table 2:** Presumptive diagnosis and whether follow-up was as advised among individuals whose skin examination revealed an abnormal finding.

	Followed up as advised		
	Total (n = 104)	Yes (n = 38)	No (n = 66)
**Diagnosis**			
Melanoma	1	1	0
Basal cell cancer	10	5	5
Melanoma + actinic keratosis	1	1	0
Squamous cell cancer + actinic keratosis	1	0	1
Basal cell cancer + actinic keratosis	5	2	3
Basal cell cancer + dysplastic nevus	3	2	1
Dysplastic nevus	15	4	11
Actinic keratosis	35	9	26
Referred, but abnormality not specified	27	7	19
Abnormality not specified/Follow-up not advised	6	6	0

Although our retention rate was excellent (90%, 283/312), it is important to determine whether those lost to follow-up had a particular pattern of skin exam results. Loss to follow-up was not more likely among those with an abnormal finding.

## 4. Discussion

Community-based screenings are designed to ensure access to expert dermatologists, particularly for those without regular access to dermatologic care. A large percentage of people attending free skin cancer screenings do not have a regular dermatologist and it is likely that many of the individuals who attend these screenings would not otherwise be screened [12].

The two underlying premises of all skin cancer screening programs are that individuals will recall and understand their screening diagnosis (abnormal finding *vs*. no abnormal finding) and that participants whose examination reveals an abnormal finding will pursue follow-up care as recommended. In this study, there was good understanding of screening outcomes. Only 12% (36/312) of participants did not recall their examination results. This relatively low level of incorrect recall is encouraging. In total, 33% (104/312) of participants were advised to seek follow-up care to an abnormal finding. However, 71% (66/93) of these individuals did not pursue additional care, which is clearly of concern. Given that patients whose screening examination revealed actinic keratoses and dysplastic nevi were the least likely to seek follow-up care, the importance of follow-up with these conditions should be stressed. It is possible that follow-up rates were low for these two conditions due to patient perception of low risk, given that these two findings do not represent a diagnosis of skin cancer.

None of the demographic, behavioral, or cognitive variables were predictive of accurate recall of examination results or follow-up status. One implication may be that no special efforts are needed to target those at greatest risk of non-compliance with screening recommendations. However, the many participants who did not seek recommended follow-up indicates that strategies are needed to increase follow-up, and suggests the need to institute mechanisms to track screenees who fail to schedule or attend scheduled appointments. Unfortunately, costs and personnel associated with maximizing follow-up efforts may result in fewer attendees being screened, and the advantages of screening many individuals may outweigh the disadvantage of not being able to ensure that every individual will follow-up.

Given the constraints of clinician’s voluntary participation and the absence of a clinical infrastructure for follow-up, the best practice for community-based screenings may be to continue to offer referrals when needed, as is currently done, and the onus for follow-up will likely remain with the participant. However, specific mechanisms must be worked out in advance of screenings to ensure that persons with presumptive diagnoses of skin cancer are contacted within a month of screening to make sure that follow-up appointments are scheduled. Screenees should receive written and verbal instructions detailing positive findings and the importance of seeing a dermatologist for follow-up from clinicians and alerting them to the American Academy of Dermatology’s toll-free number. Organizations conducting community-based screenings should consider utilizing volunteers onsite to offer referral assistance and to ensure that participants understand the importance of follow-up care. The need for strategies to increase follow-up would likely be beneficial in other countries where community-based screenings take place, especially in health care systems where screenees incur follow-up costs, which may impact the pursuit of recommended follow-up. Similar strategies such as the use of volunteers and tracking mechanisms that are tailored to a health care system would likely be beneficial for increasing in other countries where community-based screenings take place.

Message development work is needed to determine how to present results to maximize follow-up and to be sensitive to location-messages promoting action in the U.S. may not resonate elsewhere. Furthermore, as 10% of the sample pursued follow-up care that was not advised, research is also needed how best to frame the message so that fewer screenees pursue follow-up care that may not be needed. For example, research suggests that personalized risk communication, in which the risk of an outcome (e.g., skin cancer) is tailored to the individual’s behaviors and characteristics, may increase participation in screenings [25]. Thus, personalized risk communication may also promote receipt of advised follow-up after an abnormal finding during a community-based skin cancer screening. Research is needed on how to best frame these personalized messages.

This study is limited to two beaches and, at baseline, 30% of participants reported that they had previously had a full-body dermatologist skin examination, which may limit generalizability. Others have found lower rates of ever being screened for skin cancer by physicians [6,26]. The lack of significant findings in this study could be due to low statistical power or unmeasured correlates, such as perception of dermatologist's concern about screening findings. In retrospect, as part of survey 3 (the final survey), it would have been beneficial to ascertain why patients did not pursue recommended follow-up. Study strengths include a high-risk sample and participants’ diversity in education.

## 5. Conclusions

The lack of follow-up by participants with an abnormal finding is of concern, and community-based screening organizers should consider implementing improved follow-up mechanisms. Future work should explore personal motivations and delineate barriers to obtaining appropriate follow-up, and test strategies to remove barriers. Work is needed to develop messages to ensure needed follow-up is pursued, and future research should test communication messages among individuals attending community-based skin cancer screenings.
